# Systematic review and meta-analysis: the efficacy and safety of radiofrequency ablation for early superficial esophageal squamous cell neoplasia

**DOI:** 10.1186/s12876-024-03250-7

**Published:** 2024-05-02

**Authors:** Hsu-En Cheng, Sz-Iuan Shiu, Chung-Wang Ko

**Affiliations:** 1 Division of Gastroenterology and Hepatology, Department of Internal Medicine, Taichung Veterans General Hospital, No. 1650 Taiwan Boulevard Sect. 4, Taichung, 40705 Taiwan; 2 Department of Critical Care Medicine, Taichung Veterans General Hospital, No. 1650 Taiwan Boulevard Sect. 4, Taichung, 40705 Taiwan; 3 Department of Internal Medicine, Yang Ming Chiao Tung University, Taipei, Taiwan

**Keywords:** Early superficial esophageal squamous cell neoplasia, Endoscopic radiofrequency ablation, Endoscopic submucosal dissection, Meta-analysis

## Abstract

**Background and Aim:**

Esophageal squamous cell neoplasia (ESCN) is predominant in Asia. Endoscopic mucosal resection and endoscopic submucosal dissection (ESD) have both been recommended worldwide, however the application of endoscopic radiofrequency ablation (RFA) for treatment of early superficial ESCN remains inconclusive. We conducted a meta-analysis to study the effectiveness of RFA for early superficial ESCN.

**Methods:**

Three major bibliographic databases were reviewed for the enrollment of case series and cohort trials prior to August 23, 2023. We included adults diagnosed with early superficial ESCN who had been receiving endoscopic RFA or ESD if the treatments were available. Our focus was on the 12-month histological complete response rate (CR) and 3-month histological CR, as well as the acute and late postoperative adverse events (AEs) rate during the at follow-up periods.

**Results:**

Nine studies were enrolled for qualitative synthesis of narrative review, with eight trials involving a total of 447 participants for analysis. The pooled 12-month and 3-month histological CR were 0.83 (95% CI, 0.59–0.94, *I*^2^ = 80%) and 0.74 (95% CI, 0.67–0.80, *I*^2^ = 0%), respectively. As for safety, the acute and late postoperative AEs were 0.11 (95% CI, 0.05–0.26, *I*^2^ = 68%) and 0.19 (95% CI, 0.14–0.26, *I*^2^ = 0%), respectively. In subgroup analysis, the incidence of bleeding, laceration and perforation after endoscopic RFA showed 0.06, 0.06 and 0.02, respectively. When compared with ESD, RFA showed lower acute AEs and late AEs without any obvious significance.

**Conclusions:**

For early superficial ESCN, endoscopic RFA achieved both higher 12-month complete remission and late complication postoperatively when compared to 3-month histological CR and acute AEs separately, while the stricture was encountered most commonly. The choice between endoscopic RFA and ESD remains inconclusive.

**Supplementary Information:**

The online version contains supplementary material available at 10.1186/s12876-024-03250-7.

## Introduction

Esophageal cancer was the eighth most common tumor in the year 2020, resulting in the sixth most common cancer-related cause of death in the world [1], with esophageal squamous cell neoplasia (ESCN) being the predominant type of esophageal cancer [2]. The 5-year survival rate of stage I disease has been reported to be from 44.2 to 72.6% in two large population-based cohort studies [3, 4], with rate of 3.4 to 16.6% being seen for stage IV disease. Early endoscopic surveillance and intervention for ESCN have been proven to reduce both incidence of the disease and cancer-related mortality significantly [5], thus representing important components of ESCN control in the future [6].

Endoscopic resection (ER), including endoscopic mucosal resection (EMR) and endoscopic submucosal dissection (ESD), offers a minimally invasive characteristic as well as a detailed pathologic evaluation, with the European Society of Gastrointestinal Endoscopy [7] recommending that ESD may be a consideration for early superficial ESCN confined to the epithelium (m1) or the lamina propria (m2), with 5 and 10-year overall survival rates reported to be 85–95% [8] and more than 90%, respectively [9]. In terms of the comparison between ESD and treatment involving esophagectomy for pT1 ESCN, there was no difference in overall survival and recurrence-free survival [10], with ESD being associated with fewer adverse events. However, esophageal stricture after ESD may develop during long-term follow-up when encountering ESCN, with characteristics including involvement of the upper third of the esophagus, a longer longitudinal diameter and a circumferential range > 3/4 [11].

Radiofrequency ablation (RFA), which has been demonstrated as a preferred endoscopic therapy for Barrett’s esophagus in recent years [12], has also been an alternative technique for treatment of early superficial ESCN since the year 2008 [13], however the application of RFA remains inconclusive. The aim of this meta-analysis is to inspect the effectiveness of RFA as a form of treatment for early superficial ESCN, while also performing a head-to-head comparison between RFA and ESD.

## Materials and methods

### Search strategy and selection criteria

We searched various electronic databases, including PubMed, Embase and the Cochrane Central Register of Controlled Trials without any language restrictions; performing our searches in accordance with the Preferred Reporting Items for Systematic Reviews and Meta-analyses (PRISMA) 2020 statement (Table S1) [14] and registered the protocol in PROSPERO (CRD 42023429493). We also executed a manual literature search of bibliographies in retrieved articles and published reviews for eligible publications prior to August 23, 2023. A detailed description of the search strategies is provided in Table S2.

We included cohort studies or case series which evaluated both the efficacy and safety of interventions in adults (aged ≥ 18 years) diagnosed with early superficial ESCN, as confirmed according to image-enhanced endoscopy (IEE), histological evaluation, endoscopic ultrasound (EUS) and/or computed tomography (CT). We enrolled patients experiencing esophageal lesions which were completely flat (type 0-IIb), slightly elevated (type 0-IIa) or slightly depressed, according to the Paris classification of the endoscopic appearance of early gastrointestinal neoplasias [15]. IEEs were applied to any suspicious squamous dysplasia, including use of magnifying narrow-band image, chromoendoscopy with Lugol’s iodine or an I-scan, depending upon their availability in the respective hospitals. The subsequent histological evaluation of biopsy tissue reported moderate-grade squamous intraepithelial neoplasia (MGIN), high-grade squamous intraepithelial neoplasia (HGIN) or esophageal squamous cell carcinoma (ESCC) (limited to T1m2 invasion). There were no patients with either lymphadenopathy or distant metastasis via EUS and/or CT. Those patients who had received previous EMR or ESD for treatment of nodular lesions prior to RFA were also permitted to participate in the study.

We included reports enrolling RFA alone or in comparison of ESD. Reports that involved pediatric patients, pregnant women, or patients diagnosed with submucosal invasion, lymph node invasion, distant metastasis or esophageal stricture, or had a history of previous interventions other than ER, as well as those with severe concurrent comorbidities were all excluded.

### Outcome measures

For the clinical outcome parameters, we determined the 12-month histological complete response (CR), 3-month histological CR, acute and late postoperative adverse events (AEs), as well as the separate complication rate, including bleeding, lacerations, perforations or strictures at the follow-up periods after completion of the interventions. We defined the histological CR if IEE, including Lugol’s chromoendoscopy, yielded no Lugol’s unstained areas and when biopsies taken from the treatment area or from Lugol’s unstained areas showed the absence of squamous intraepithelial neoplasia, or ESCC at the follow-up periods. Acute postoperative AEs were subjectively judged by endoscopists if bleeding, lacerations or perforations occurred. Late postoperative AEs were recorded if stricture occurred. Ethical approval or informed consent from the participants was not necessary as there was no individual participant data involved.

### Data extraction and quality assessment

Two investigators (C-HE and S-SI) independently screened the titles and abstracts for eligibility, and full texts were assessed to clarify the eligibility status of each article. All discrepancies were discussed and later resolved upon consultation with a third investigator (K-CW). Two reviewers (C-HE and S-SI) extracted data independently, with the data then checked by a third investigator (K-CW). The following variables were extracted: country of study, participants’ characteristics, inclusion criteria, details of RFA protocol, endoscopic sedation, follow-up strategy, postoperative care and outcome measurements.

Two investigators (C-HE and S-SI) independently evaluated the risk of bias of all studies, while also assessing the quality of the articles included in the analysis through use of the Newcastle-Ottawa Scale (NOS) assessment tool [16]. Any disagreements were discussed until a consensus was reached, with a third investigator (K-CW) being consulted when necessary.

### Data synthesis and statistical analysis

The results were analyzed using R Project for Statistical Computing V.4.2.3 software and Review Manager V.5.3 software (Nordic Cochrane Centre, Copenhagen, Denmark). The pooled incidence and 95% confidence interval (CI) were reported for continuous variables, which consisted of 12-month histological CR, 3-month histological CR, acute and late postoperative AEs and separate complication rate. The pooled risk ratios (RRs) and 95% CI were reported for both binary variables and dichotomous variables. When we noted zero events, no imputation for zero cell counts with 0.5 was performed. An RR with a 95% CI was used to present the 12, and 3-month histological CRs, as well as the acute and late postoperative AEs between the comparison of RFA and ESD. These were completely produced using a random effect model to allow for the expected heterogeneity amongst the enrolled studies.

Heterogeneity of the outcome measures was examined using the Cochrane *I*^2^ statistic. We regarded an *I*^2^ of less than 25% as mild heterogeneity, 25–50% as moderate heterogeneity, and higher than 50% as severe heterogeneity. If the *x*^2^ test revealed *p* > 0.10 it was not considered significant in the heterogeneity test of the research. We checked for publication bias by carrying out visual inspection of the funnel plot, while also performing sensitivity analysis, where one study was excluded at a time in order to evaluate the between-study heterogeneity.

### Subgroup analysis

In addition, we performed subgroup analysis in order to determine the pooled outcome parameters in patients who had or had not experienced a previous ER at a male percentage rate > 50%.

## Results

**Fig. 1:**
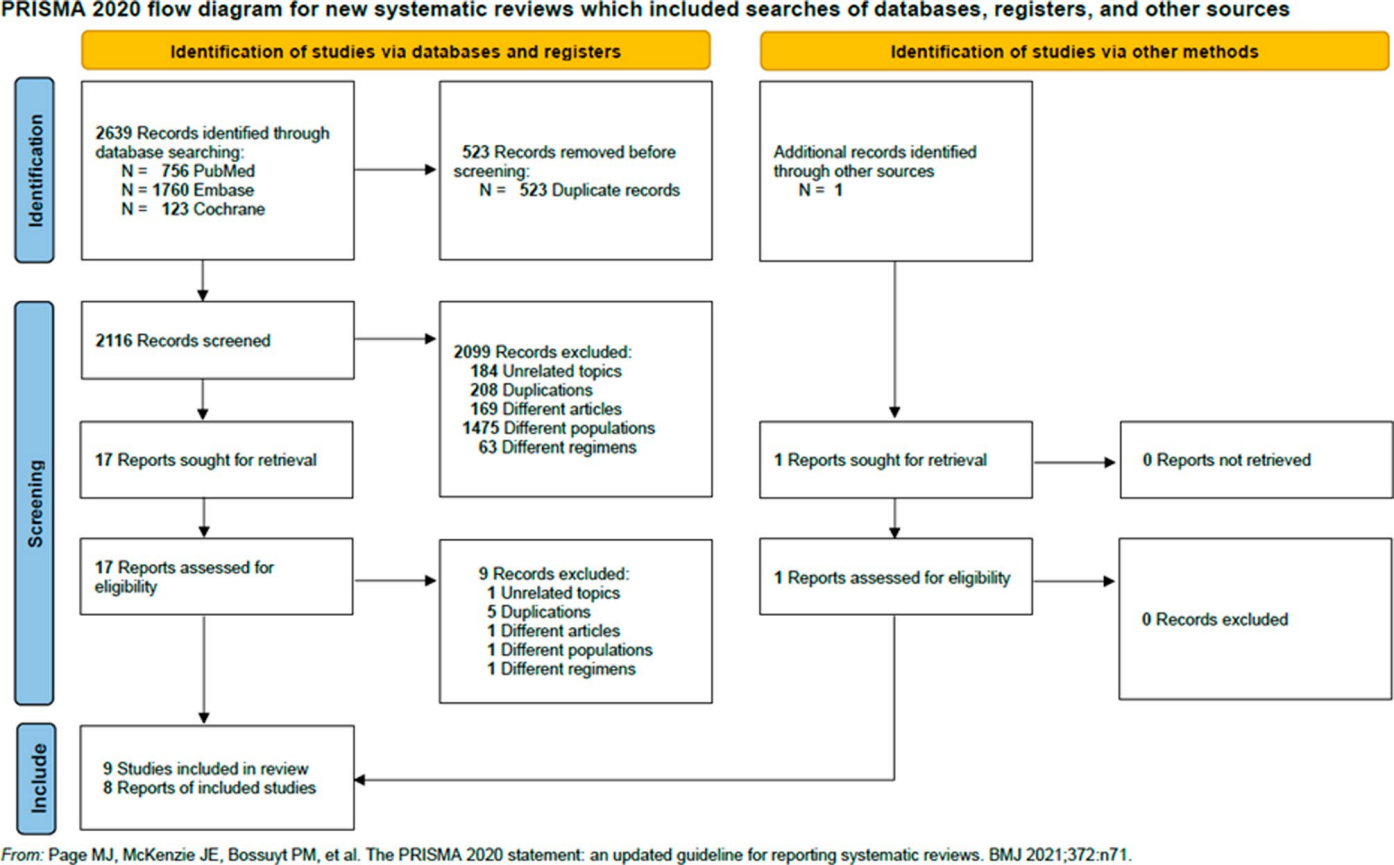
PRISMA 2020 flow diagram

After primary screening of the titles and abstracts, 18 full-text articles were assessed for eligibility (Fig. 1). Ultimately, we included 9 articles [17–25] for qualitative and 8 articles [17, 19–25] for quantitative analysis, with a total of 447 participants summarized in the supplementary reference.

### Characteristics of the included studies

The methodology and characteristics of the study design and patient outcomes of 2 prospective and 5 retrospective cohort trials plus 2 case series, are summarized in Tables S3, S4 and S5. Among these studies sample sizes ranged from 6 to 105 (median 35), while the ranges for age, percentage of male gender, previous ER rate and length of unstained lesions were 52.3–71.6, 20.0-100.0, 0-69.2 and 3.6–9.4, respectively. Most studies were performed in Taiwan and China (66.7%) with the remaining studies originating from the Netherlands, Germany and the United Kingdom. We included 3 comparative trials between RFA and ESD in our review [21, 24, 25]. The baseline patient characteristics between RFA and ESD did not showed any significant differences in the Chou et al. [24] study, while patients treated with RFA tended to have a larger tumor size [25], wider circumferential distribution [21, 25] and wider tumor location [25] as shown in the other two studies.

### Outcome parameters: efficacy and safety

**Fig. 2:**
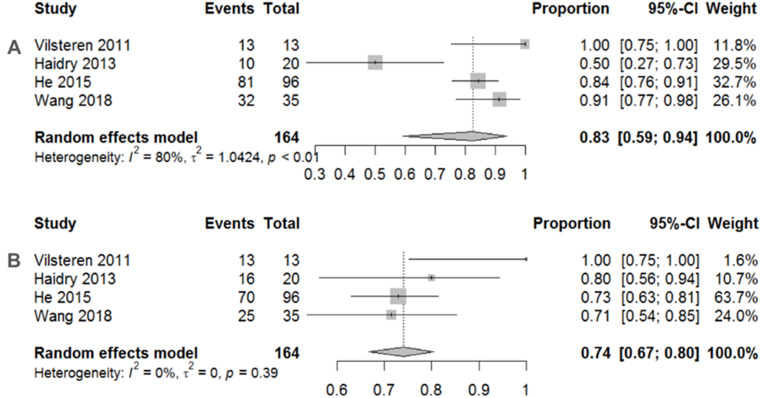
**A** Pooled incidence of 12-month histological complete remission after endoscopic radiofrequency ablation. **B** Pooled incidence of 3-month histological complete remission after endoscopic radiofrequency ablation

**Fig. 3:**
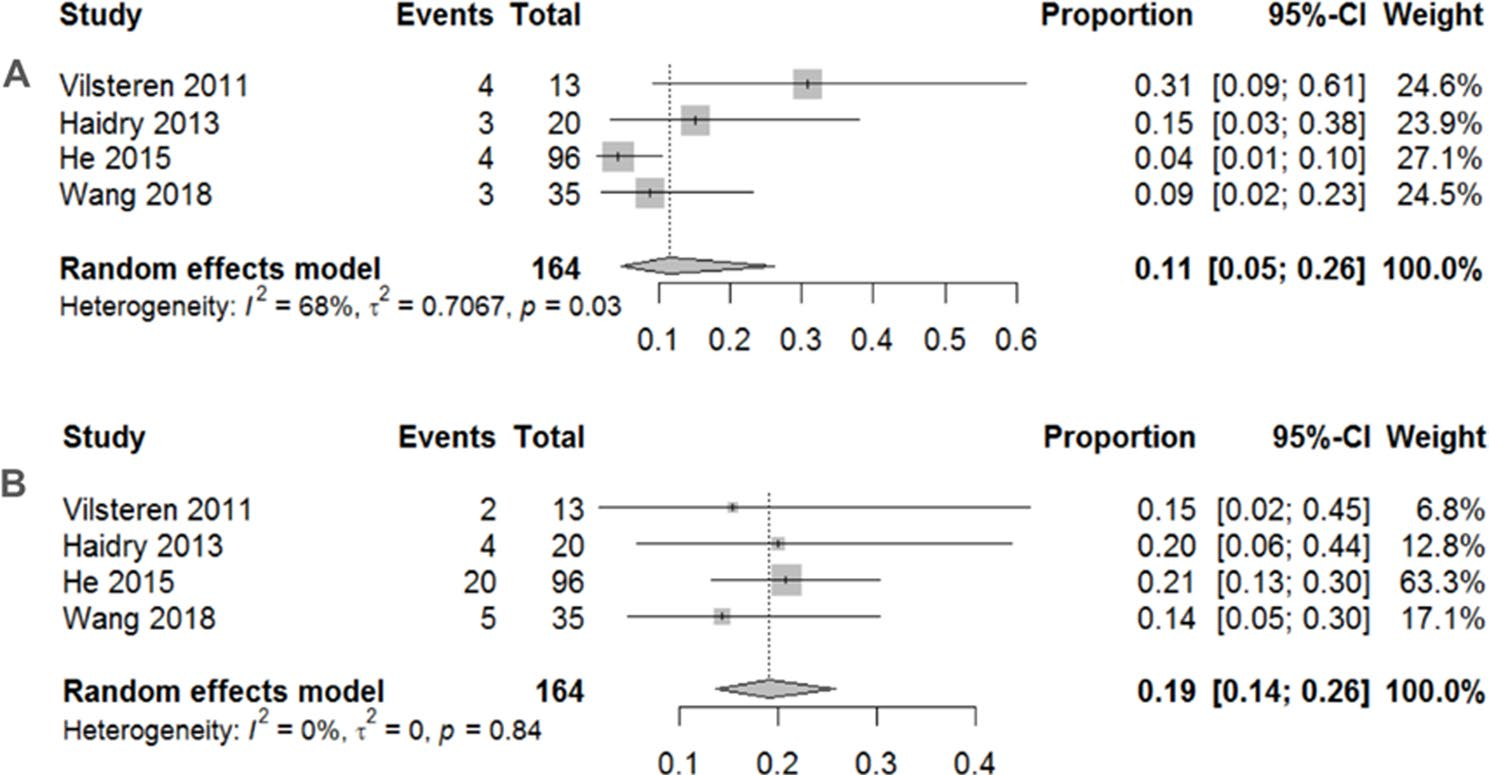
**A** Pooled incidence of acute postoperative adverse events after endoscopic radiofrequency ablation. **B** Pooled incidence of late postoperative adverse events after endoscopic radiofrequency ablation

**Fig. 4:**
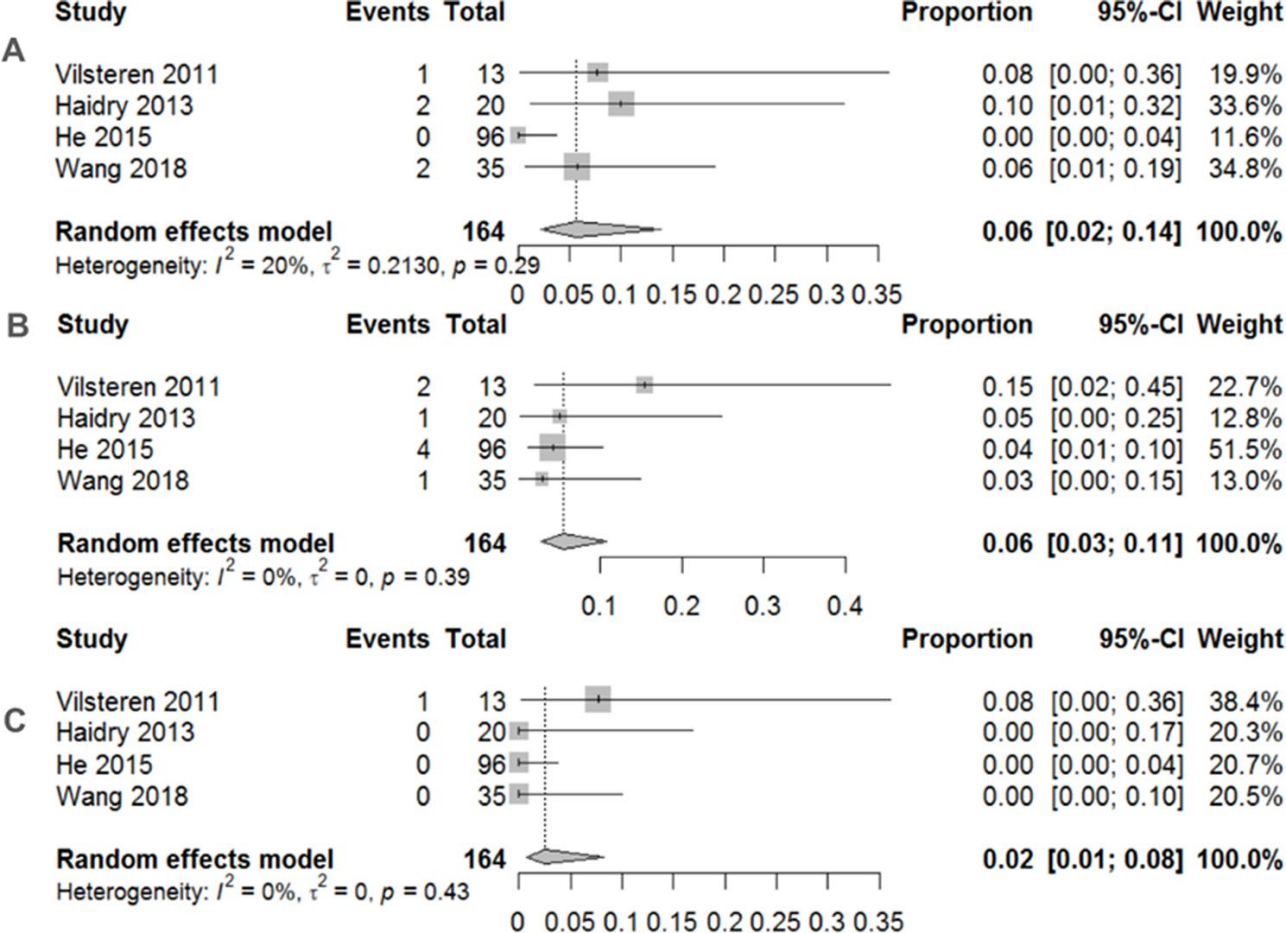
**A** Pooled complication rate of bleeding after endoscopic radiofrequency ablation. **B** Pooled complication rate of laceration after endoscopic radiofrequency Ablation. **C** Pooled complication rate of perforation after endoscopic radiofrequency ablation

Traditional meta-analyses of the included trials are shown in Figs. 2, 3 and 4. Regarding the 12-month histological CR, RFA achieved a pooled incidence of 0.83 (95% CI: 0.59–0.94, *I*^2^ = 80%, *p* < 0.01) (Fig. 2A), while RFA showed 0.74 (95% CI: 0.67–0.80, *I*^2^ = 0%, *p* = 0.39) in 3-month histological CR (Fig. 2B). As for safety issues, acute and late postoperative AEs were 0.11 (95% CI: 0.05–0.26, *I*^2^ = 68%, *p* = 0.03) (Fig. 3A) and 0.19 (95% CI: 0.14–0.26, *I*^2^ = 0%, *p* = 0.84) (Fig. 3B), respectively. Separate complication rates, including bleeding, lacerations and perforations were 0.06 (95% CI: 0.02–0.14, *I*^2^ = 20%, *p* = 0.29) (Fig. 4A), 0.06 (95% CI: 0.03–0.11, *I*^2^ = 0%, *p* = 0.39) (Fig. 4B) and 0.02 (95% CI: 0.01–0.08, *I*^2^ = 0%, *p* = 0.43) (Fig. 4C), respectively.

**Fig. 5:**
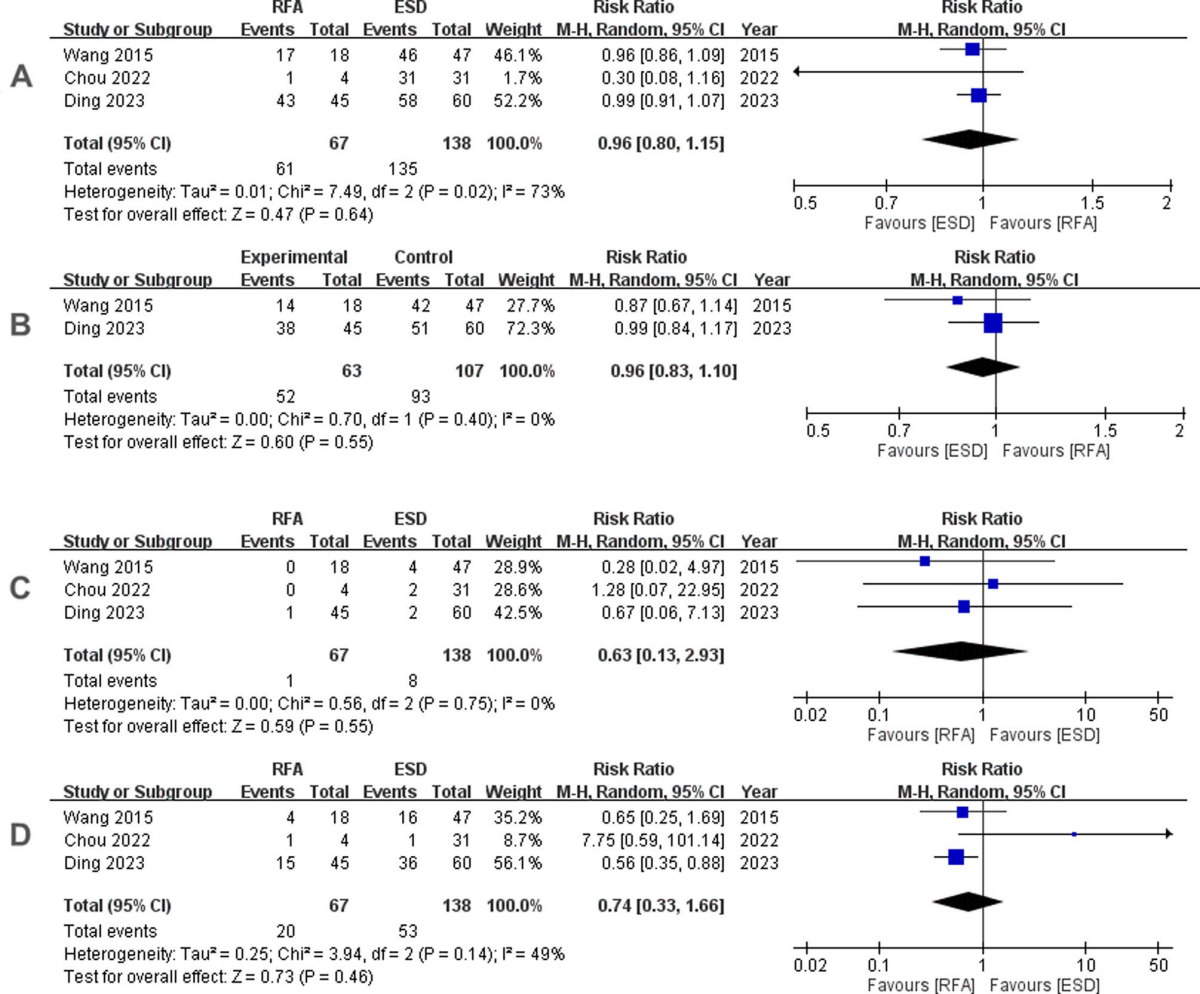
**A** Forest plot of pooled RRs between radiofrequency ablation and endoscopic submucosal dissection (12-month complete remission). **B** Forest plot of pooled RRs between radiofrequency ablation and endoscopic submucosal dissection (3-month complete remission). **C** Forest plot of pooled RRs between radiofrequency ablation and endoscopic submucosal dissection (acute postoperative adverse events). **D** Forest plot of pooled RRs between radiofrequency ablation and endoscopic submucosal dissection (late postoperative adverse events)

Additionally, there was no difference seen between RFA and ESD in 12-month histological CR (RR 0.96, 95% CI: 0.80–1.15, *I*^2^ = 73%, *p* = 0.64) (Fig. 5A), 3-month histological CR (RR 0.96, 95% CI: 0.83–1.10, *I*^2^ = 0%, *p* = 0.55) (Fig. 5B), acute postoperative AEs (RR 0.63, 95% CI: 0.13–2.93, *I*^2^ = 0%, *p* = 0.55) (Fig. 5C), or late postoperative AEs (RR 0.74, 95% CI: 0.33–1.66, *I*^2^ = 49%, *p* = 0.46) (Fig. 5D). The funnel plots of this meta-analysis could not be judged due to the inclusion of less than 5 trials in each comparative outcome. Due to the higher heterogeneity in the 12-month histological CR and late postoperative AEs, we conducted sensitivity analysis by excluding one study [24] in order to assess the between-study heterogeneity. The results showed there was an improvement in both the heterogeneity of the 12-month histological CR (RR 0.98, 95% CI: 0.92–1.05, *I*2 = 0%, *p* = 0.57) and late postoperative AEs (RR 0.57, 95% CI: 0.38–0.87, *I*2 = 0%, *p* < 0.01), with statistical significance being seen in late postoperative AEs.

**Fig. 6:**
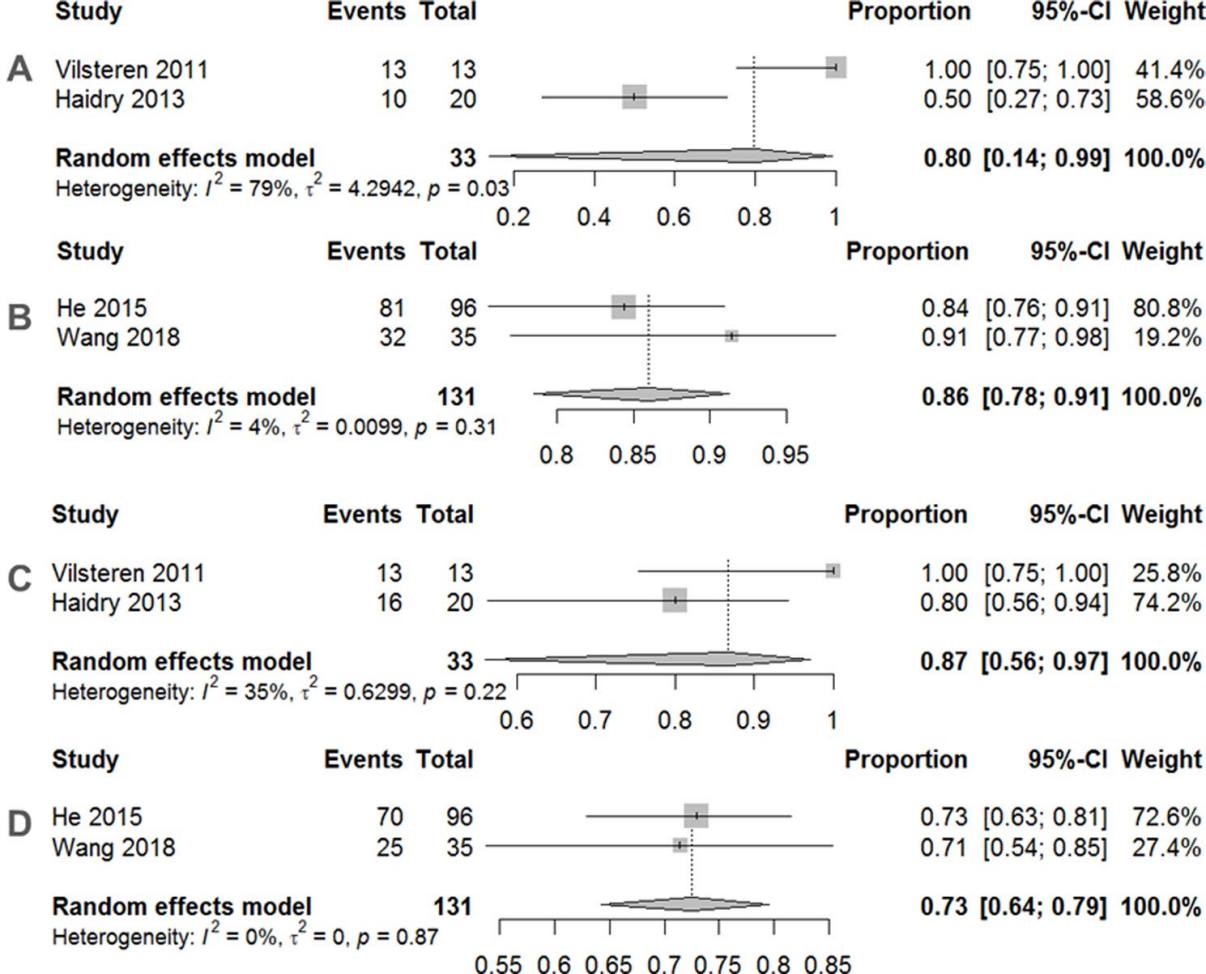
**A** Pooled incidence of 12-month histological complete remission after endoscopic radiofrequency ablation with previous ER. **B** Pooled incidence of 12-month histological complete remission after endoscopic radiofrequency ablation without previous ER. **C** Pooled incidence of 3-month histological complete remission after endoscopic radiofrequency ablation with previous ER. **D** Pooled incidence of 3-month histological complete remission after endoscopic radiofrequency ablation without previous ER

**Fig. 7:**
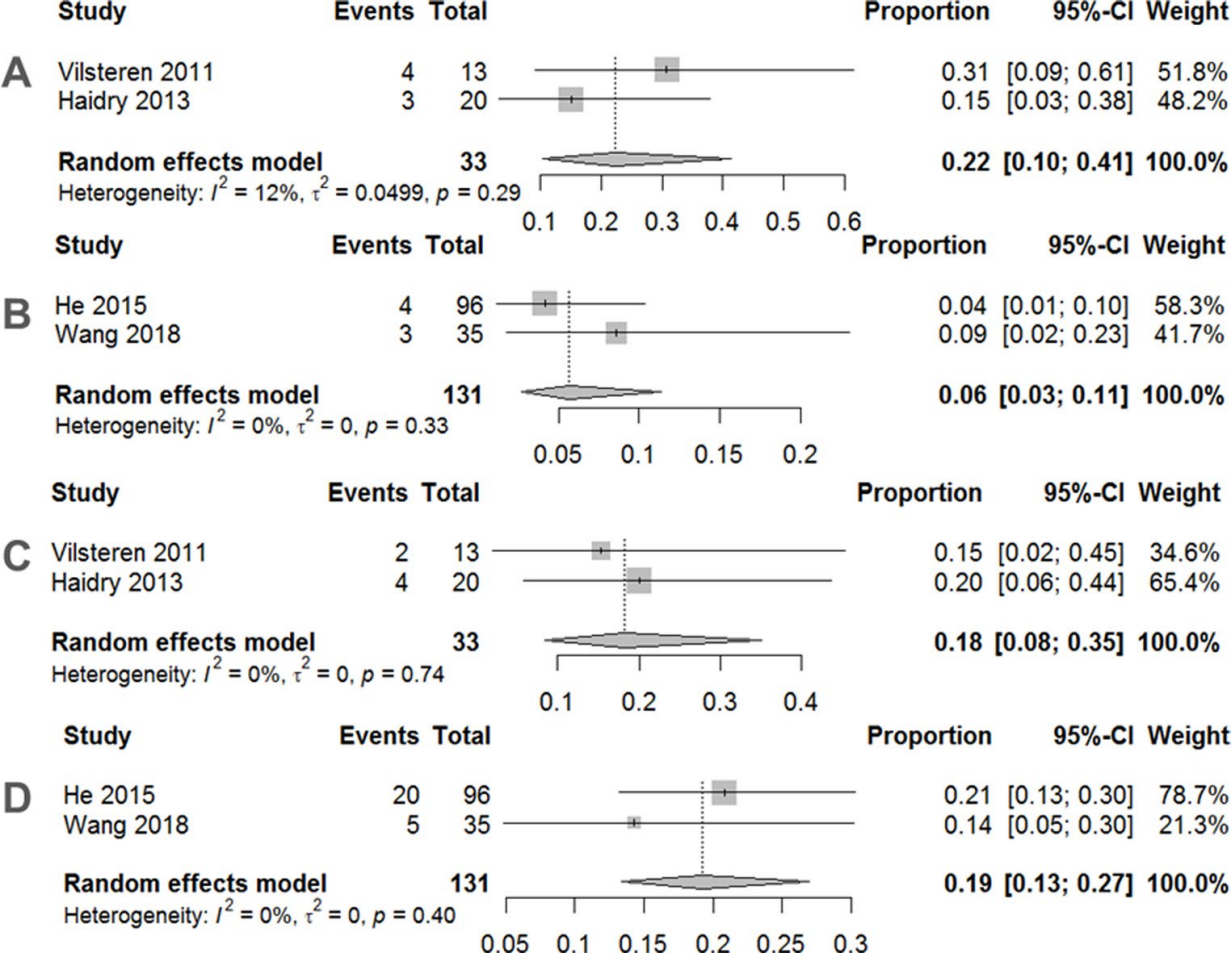
**A** Pooled incidence of acute postoperative adverse events after endoscopic radiofrequency ablation with previous ER. **B** Pooled incidence of acute postoperative adverse events after endoscopic radiofrequency ablation without previous ER. **C** pooled incidence of late postoperative adverse events after endoscopic radiofrequency ablation with previous ER. **D** Pooled incidence of late postoperative adverse events after endoscopic radiofrequency ablation without previous ER. *Abbreviations RRs* relative risks, *CI* confidence interval

In subgroup analysis, we demonstrated that the 12-month histological CR of RFA for those with a previous ER (0.80, 95% CI: 0.14–0.99, *I*^2^ = 79%, *p* = 0.03) (Fig. 6A) was similar to that of RFA without a previous ER (0.86, 95% CI: 0.78–0.91, *I*^2^ = 4%, *p* = 0.31) (Fig. 6B). Meanwhile, the 3-month histological CR of RFA for those with a previous ER (0.87, 95% CI: 0.56–0.97, *I*^2^ = 35%, *p* = 0.22) (Fig. 6C) was better than that of RFA without a previous ER (0.73, 95% CI: 0.64–0.79, *I*^2^ = 0%, *p* = 0.87) (Fig. 6D). As for acute postoperative AEs, RFA for those with a previous ER (0.22, 95% CI: 0.10–0.41, *I*^2^ = 12%, *p* = 0.29) (Fig. 7A) was inferior to RFA without a previous ER (0.06, 95% CI: 0.03–0.11, *I*^2^ = 0%, *p* = 0.33) (Fig. 7B). When considering late postoperative AEs, RFA for those with a previous ER (0.18, 95% CI: 0.08–0.35, *I*^2^ = 0%, *p* = 0.74) (Fig. 7C) was similar to RFA without a previous ER (0.19, 95% CI: 0.13–0.27, *I*^2^ = 0%, *p* = 0.40) (Fig. 7D).

In trials having a male percentage > 50%, the 12-month histological CR was 0.87 (95% CI: 0.79–0.92, *I*^2^ = 4%, *p* = 0.35) (Figure S1A), while the 3-month histological CR, acute postoperative AEs and late postoperative AEs were 0.74 (95% CI: 0.63–0.82, *I*^2^ = 23%, *p* = 0.27) (Figure S1B), 0.11 (95% CI: 0.03–0.32, *I*^2^ = 77%, *p* = 0.01) (Figure S1C) and 0.19 (95% CI: 0.13–0.26, *I*^2^ = 0%, *p* = 0.66) (Figure S1D), respectively.

### Risk of bias assessment

The NOS assessment tool is outlined in Table S6, encompassing eight trials from our meta-analysis. The scores in most of the trials were less than 7 points considering to be of poor quality. Factors such as the absence of selection of the non-exposed cohort, lack of adjustment for confounders, and insufficient follow-up of cohorts contributed to downgrading in each respective domain and led to a zero-star rating in the comparability domain.

## Discussion

In this meta-analysis, we comprehensively evaluated the effectiveness of RFA in patients diagnosed with early superficial ESCN. We have demonstrated that endoscopic RFA achieved both a higher 12-month complete remission, as well as late complication postoperatively. With respect to safety, stricture was encountered most commonly, followed by bleeding/laceration, and perforation. In subgroup analysis, the 3-month histological CR of RFA for those with a previous ER proved to be better than that of RFA without a previous ER, while acute postoperative AEs of RFA for those with a previous ER was inferior to that of RFA without a previous ER. As for 12-month histological CR and late postoperative AEs, RFA for those with a previous ER was similar to RFA without a previous ER. In addition, the choice between endoscopic RFA and ESD remains inconclusive.

According to the American guidelines established in both 2016 and 2020 [26, 27], RFA has been recommended for BE with high-grade dysplasia or T1a esophageal adenocarcinoma, as well as low-grade dysplasia. However, whether RFA should be recommended as the first priority for early superficial ESCC remains inconclusive. A previous review [28] reported that the overall CR within 12 months varied from 50 to 100%, but these studies involved primarily small cohorts with sample sizes of less than 30, along with discrepant study protocols. Although pretreatment tumor staging via biopsy histology can predict the local recurrence of ESCN independently [22], determining the appropriate surveillance interval is crucial. In our review, the follow-up period ranged from 1 to 3 months after the procedure, and subsequently every 3 to 6 months for 1 year thereafter, as recommended by expert opinion. Notably, the factors related to local recurrence were different between these two follow-up periods. The major determinants of 3-month histological CR are pretreatment tumor staging and the prescribed regimen for RFA. The pink-color sign [23], ductal invasion [22, 29] and length of unstained lesions [20] are all risk factors associated with local recurrence after successful RFA, with Jansen et al. having reported that 35% of superficial ESCN eligible for ablation through endoscopic image review were histologically underestimated [30]. Therefore, ensuring accurate pre-procedure staging and careful patient selection are essential when considering RFA as a treatment option.

Moreover, there is currently no consensus regarding the standard protocol surrounding RFA for ESCN, which may introduce mutual confounders in both 3-month and 12-month histological CR. Two studies [31, 32] using balloon-based radiofrequency ablation for patients with BE demonstrated that two applications of 10–12 J/cm^2^ together with intermediate cleaning achieved better regression and enhanced ablation energy if cleaned immediately after the first application. This form of treatment has also been adopted as a common practice for circumferential RFA in ESCN. However, for focal RFA, a regimen of one to three applications of 12 J/cm^2^ has been widely used without any trials when comparing the dose-response relationship among these regimens. In our study, we observed a 3-month histological CR of 0.74 after RFA, while patients with previous ER demonstrated an even higher 3-month CR of 0.87, thus indicating that the combination of RFA with ER for ESCN was encouraging. Further validation through the use of large-scale trials is awaiting so as to better establish its efficacy more definitively.

The surveillance programs for 12-month histological CR differed from the enrolled studies which encompassed variations in follow-up intervals, screening modalities and salvage treatments based on the stage of recurrence. In our meta-analysis, the overall 12-month CR was 0.83, which was higher as compared to the 3-month histological CR, but considerable variability existed among the included studies. In a study conducted by Vilsteren et al. [17] a 100% histological CR was achieved during a median follow-up period of 17 months. The study had enrolled only 13 patients, of whom 69% underwent ER prior to undergoing RFA. According to the report by He et al. [20] a total of 96 patients without any prior ER were enrolled in their study, which achieved an 84.4% histological CR at 12 months following additional RFA. In contrast, Haidry et al. [19] reported a 12-month histological CR of 50% in a cohort where a single application of 12 J/cm^2^ was utilized. Furthermore, the 12-month histological CR showed consistency between subgroup analyses of patients without a previous ER, as well as those having had a previous ER.

With respect to safety, the most common AE was stricture, followed by bleeding, laceration, and perforation, with a pooled incidence of 0.19, 0.06, 0.06 and 0.02, respectively. A meta-analysis [11] involving 11 studies with 2,248 participants reported a pooled stricture after ESD to be 12.2%, thus indicating that 6 substantial risk factors were related to an elevated risk of post-ESD esophageal stricture. The stricture occurrence would increase to 70–80% if the extent of circumferential resection became > 60% of esophageal circumferences [33]. The incidence of stricture after RFA for ESCN is relatively low when compared to that after ESD, with possible mechanisms to elucidate increased stricture formation after ESD including withering of the muscularis propria [34], fibrosis of the submucosa [35], muscle layer damage [36], unsatisfactory hemostasis [37] and longer coagulation time [38]. In contrast, RFA has the advantage of both minimizing damage to the muscularis propria and reducing thermal damage after hemostasis, ultimately preventing RFA-related stenosis. Furthermore, our findings suggest that a previous ER may not be associated with stenosis, which contradicts the results of a previous study [17], while a previous ER could potentially reduce acute postoperative AEs following RFA.

According to the Japanese Esophageal Society and the European Society for Medical Oncology guidelines [39, 40], EMR/ESD is recommended for ESCN treatment in groups of patients with T1a cancer above the muscularis mucosae with a non-circumferential lesion, or for T1a cancer above the lamina propria mucosae with a circumferential lesion of less than 5 cm for en-bloc resection. Although RFA has been gradually applied to early superficial ESCN, the comparison between these two modalities has been limited. We enrolled only 3 trials [21, 24, 25], which precluded the funnel plots of this meta-analysis and indicated the both existence of selection bias and publication bias. The patients treated with RFA tended to have a longer sized and larger circumferential extension of the tumor than those treated with ESD [21, 25]. The choice between endoscopic RFA and ESD remains unsettled based on a very low quality of evidence.

There are several limitations to this meta-analysis. Firstly, the potential existence of publication bias and methodological bias in our review should be acknowledged. We refrained from conducting funnel plots due to the limited number of trials in each comparative outcome and observed variations in the protocol of RFA for ESCN. Although, in comparison to a previous review [28], five out of seven studies in our review had a sample size of more than 30 participants, thus making it important to recognize the constraints owing to limited sources. Conducting further prospective studies involving multiple centers would be beneficial towards enhancing the applicability. Secondly, we primarily obtained short-term outcomes of histological CR of up to 12 months with a scarcity of long-term disease-free outcomes. Yu et al. [23] reported a 5-year histological CR of 85.9% and a late esophageal stricture of 25.6% in a cohort of patients who underwent RFA for ESCN. In comparison, for ESCN treated with ESD, a Japanese cohort study [8] demonstrated a 5-year overall survival rate of 85–95%, and a Taiwanese cohort study [9] reported a 10-year disease-free survival rate of more than 90%. Thirdly, we did not perform meta-regression according to tumor size, circumference or protocol of RFA in order to examine the impact of patient variables on trial outcomes due to missing characteristics. Instead, we performed subgroup analysis in patients with a previous ER history and sensitivity analyses after omitting articles which detected outliers in order to explore the impact of heterogeneity in our sample population. The Chou et al. study [24] enrolled only 4 patients in its RFA group, which was less than the other two studies, with any sampling error due to a small sample size possibly being considered in our meta-analysis. Finally, zero stars were common in comparability domain due to non-adjustment for confounders in most trials. Moreover, variability in baseline characteristics, surveillance programs, and outcomes measurements assaulted the transitivity of meta-analysis and rendered clinical outcomes unreliable especially clinical remission and adverse events. All of the above limitations indicate that our results are based on a poor quality of evidence.

## Conclusions

For early superficial ESCN, endoscopic RFA achieved a higher mid-term efficacy and late complication postoperatively, while a stricture was most commonly encountered. There was no difference seen between RFA and ESD concerning both efficacy and safety as this was based on a very low quality of evidence. Our meta-analysis may be of value to clinicians, as the findings suggest that endoscopic RFA may be considered as an alternative option beyond the recommendations provided by the current guidelines.

### Electronic supplementary material

Below is the link to the electronic supplementary material.

Supplementary Material 1

## Data Availability

All data relevant to the study has either been included in the article or been uploaded as supporting information.

## Declarations

### Patient and public involvement

Patients and/or the public were not involved in the design, conduct, reporting or dissemination plans of this research.

### Provenance and peer review

Not commissioned; externally peer reviewed.
