# Supplementary material for: Systematic review and meta-analysis: the efficacy and safety of radiofrequency ablation for early superficial esophageal squamous cell neoplasia
Source: BMC Gastroenterol. 2024 May 2;24:152. doi: 10.1186/s12876-024-03250-7 (PMC11067246; doi:10.1186/s12876-024-03250-7)
Supplement: Supplementary file 1 — Supplementary Material 1 [file 12876_2024_3250_MOESM1_ESM.doc]

**Supplementary Content Page(s)**

Table 1. PRISMA Extension Checklist 2-5

Table 2. Electronic Database Searching Strategy 6

Table 3. Narration of Enrolled Cohort Trials 7

Table 4. Technical Characteristics of Enrolled Cohort Trials 8

Table 5. Outcomes Characteristics of Enrolled Cohort Trials 9-10

Table 6. Risk of Bias for Cross sectional Trials by Newcastle-Ottawa Scale Tool

11

Figure 1A. Pooled Incidence of 12-month Histological Complete Remission after Endoscopic Radiofrequency Ablation (Male percentage > 50%) 12

Figure 1B: Pooled Incidence of 3-month Histological Complete Remission after Endoscopic Radiofrequency Ablation (Male percentage > 50%) 12

Figure 1C: Pooled Incidence of Acute Postoperative Adverse Events after Endoscopic Radiofrequency Ablation (Male percentage > 50%)

Figure 1D: Pooled Incidence of Late Postoperative Adverse Events after Endoscopic Radiofrequency Ablation (Male percentage > 50%)Supplementary References 13

**Supplementary Table 1. PRISMA Extension Checklist^1^**

| **Section/Topic** | **Item #** | **Checklist item** | **Reported on Page #** |
| --- | --- | --- | --- |
| **TITLE** | | |  |
| Title | 1 | Identify the report as a systematic review. | Title page |
| **ABSTRACT** | | |  |
| Abstract | 2 | See the PRISMA 2020 for Abstracts checklist. | Page 3-4 |
| **INTRODUCTION** | | |  |
| Rationale | 3 | Describe the rationale for the review in the context of existing knowledge. | Page 5 |
| Objectives | 4 | Provide an explicit statement of the objective(s) or question(s) the review addresses. | Page 5 |
| **METHODS** | | |  |
| Eligibility criteria | 5 | Specify the inclusion and exclusion criteria for the review and how studies were grouped for the syntheses. | Page 6 |
| Information sources | 6 | Specify all databases, registers, websites, organizations, reference lists and other sources searched or consulted to identify studies. Specify the date when each source was last searched or consulted. | Page 6 |
| Search strategy | 7 | Present the full search strategies for all databases, registers and websites, including any filters and limits used. | Page 6  Table S2 |
| Selection process | 8 | Specify the methods used to decide whether a study met the inclusion criteria of the review, including how many reviewers screened each record and each report retrieved, whether they worked independently, and if applicable, details of automation tools used in the process. | Page 7 |
| Data collection process | 9 | Specify the methods used to collect data from reports, including how many reviewers collected data from each report, whether they worked independently, any processes for obtaining or confirming data from study investigators, and if applicable, details of automation tools used in the process. | Page 7 |
| Data items | 10a | List and define all outcomes for which data were sought. Specify whether all results that were compatible with each outcome domain in each study were sought (e.g., for all measures, time points, analyses), and if not, the methods used to decide which results to collect. | Page 6 |
|  | 10b | List and define all other variables for which data were sought (e.g., participant and intervention characteristics, funding sources). Describe any assumptions made about any missing or unclear information. | Page 7 |
| Study risk of bias assessment | 11 | Specify the methods used to assess risk of bias in the included studies, including details of the tool(s) used, how many reviewers assessed each study and whether they worked independently, and if applicable, details of automation tools used in the process. | Page 7 |
| Effect measures | 12 | Specify for each outcome the effect measure(s) (e.g., risk ratio, mean difference) used in the synthesis or presentation of results. | Page 7 |
| Synthesis methods | 13a | Describe the processes used to decide which studies were eligible for each synthesis (e.g., tabulating the study intervention characteristics and comparing against the planned groups for each synthesis (item #5)). | Page 7 |
|  | 13b | Describe any methods required to prepare the data for presentation or synthesis, such as handling of missing summary statistics, or data conversions. | Page 7-8 |
|  | 13c | Describe any methods used to tabulate or visually display results of individual studies and syntheses. | Page 7-8 |
|  | 13d | Describe any methods used to synthesize results and provide a rationale for the choice(s). If meta-analysis was performed, describe the model(s), method(s) to identify the presence and extent of statistical heterogeneity, and software package(s) used. | Page 7-8 |
|  | 13e | Describe any methods used to explore possible causes of heterogeneity among study results (e.g., subgroup analysis, meta-regression). | Page 8 |
|  | 13f | Describe any sensitivity analyses conducted to assess robustness of the synthesized results. | Nil |
| Reporting bias assessment | 14 | Describe any methods used to assess risk of bias due to missing results in a synthesis (arising from reporting biases). | Nil |
| Certainty assessment | 15 | Describe any methods used to assess certainty (or confidence) in the body of evidence for an outcome. | Nil |
| **RESULTS** | | |  |
| Study selection | 16a | Describe the results of the search and selection process, from the number of records identified in the search to the number of studies included in the review, ideally using a flow diagram. | Page 8  Figure 1 |
|  | 16b | Cite studies that might appear to meet the inclusion criteria, but which were excluded, and explain why they were excluded. | Nil |
| Study characteristics | 17 | Cite each included study and present its characteristics. | Page 8  Table S3,S4,S5 |
| Risk of bias in studies | 18 | Present assessments of risk of bias for each included study. | Table S6,S7 |
| Results of individual studies | 19 | For all outcomes, present, for each study: (a) summary statistics for each group (where appropriate) and (b) an effect estimates and its precision (e.g., confidence/credible interval), ideally using structured tables or plots. | Page 9  Figure 2,3,4 |
| Results of syntheses | 20a | For each synthesis, briefly summarize the characteristics and risk of bias among contributing studies. | Page 9 |
|  | 20b | Present results of all statistical syntheses conducted. If meta-analysis was done, present for each the summary estimate and its precision (e.g., confidence/credible interval) and measures of statistical heterogeneity. If comparing groups, describe the direction of the effect. | Page 9 |
|  | 20c | Present results of all investigations of possible causes of heterogeneity among study results. | Page 9-10 |
|  | 20d | Present results of all sensitivity analyses conducted to assess the robustness of the synthesized results. | Nil |
| Reporting biases | 21 | Present assessments of risk of bias due to missing results (arising from reporting biases) for each synthesis assessed. | Nil |
| Certainty of evidence | 22 | Present assessments of certainty (or confidence) in the body of evidence for each outcome assessed. | Nil |
| **DISCUSSION** | | |  |
| Discussion | 23a | Provide a general interpretation of the results in the context of other evidence. | Page 10 |
|  | 23b | Discuss any limitations of the evidence included in the review. | Page 11-12 |
|  | 23c | Discuss any limitations of the review processes used. | Page 11-12 |
|  | 23d | Discuss implications of the results for practice, policy, and future research. | Page 12 |
| **OTHER INFORMATION** | | |  |
| Registration and protocol | 24a | Provide registration information for the review, including register name and registration number, or state that the review was not registered. | Page 6 |
|  | 24b | Indicate where the review protocol can be accessed, or state that a protocol was not prepared. | Nil |
|  | 24c | Describe and explain any amendments to information provided at registration or in the protocol. | Nil |
| Support | 25 | Describe sources of financial or non-financial support for the review, and the role of the funders or sponsors in the review. | Title page |
| Competing interests | 26 | Declare any competing interests of review authors. | Title page |
| Availability of data, code and other materials | 27 | Report which of the following are publicly available and where they can be found: template data collection forms; data extracted from included studies; data used for all analyses; analytic code; any other materials used in the review. | Title page |

PICOS, population, intervention, comparators, outcomes, study design.

**PICOS Statement**

Population: The patient population is adults (aged ≥ 18 years) with early superficial esophageal squamous cell neoplasia (ESCN) which was confirmed according to image-enhanced endoscopy, histological evaluation, endoscopic ultrasound and/or computed tomography.

Intervention:

We compared the relative efficacy and safety of early superficial ESCN treatments as follows:

RFA: radiofrequency ablation.

Comparator if available:

ESD: endoscopic submucosal dissection.

Outcome:

12-months histological complete response (CR), 3-months histological CR, acute and late postoperative adverse events, and separate complication rate including bleeding, laceration, perforation, or stricture.

**Supplementary Table 2. Electronic Database Searching Strategy**

| **PubMed searching strategy** | | |
| --- | --- | --- |
| Population / Interventions | #1 | (((Esophageal cancer[MeSH Terms]) OR (Esophageal neoplasm[MeSH Terms]) OR (Esophagus[Title/Abstract] OR Esophageal[Title/Abstract] OR Oesophagus[Title/Abstract] OR Oesophageal[Title/Abstract])) AND (Cancer[Title/Abstract] OR Neoplasm[Title/Abstract] OR Malignancy[Title/Abstract] OR Carcinoma[Title/Abstract] OR Dysplasia[Title/Abstract] OR Dysplastic[Title/Abstract] OR Squamous[Title/Abstract] OR LGD[Title/Abstract] OR HGD[Title/Abstract] OR CIS[Title/Abstract] OR SCC[Title/Abstract]))  AND  ((Radiofrequency catheter ablation[MeSH Terms]) OR (Radiofrequency[Title/Abstract] AND (Ablation[Title/Abstract] OR Energy[Title/Abstract])) OR RFA[Title/Abstract] OR BARRX[Title/Abstract]) |
| **Embase searching strategy** | | |
| Population / Interventions | #1 | ('esophagus cancer'/exp) OR ('esophagus tumor'/exp) OR ((esophagus:ti,ab,kw OR esophageal:ti,ab,kw OR oesophagus:ti,ab,kw OR oesophageal:ti,ab,kw) AND (cancer:ti,ab,kw OR neoplasm:ti,ab,kw OR malignancy:ti,ab,kw OR carcinoma:ti,ab,kw OR dysplasia:ti,ab,kw OR dysplastic:ti,ab,kw OR squamous:ti,ab,kw OR LGD:ti,ab,kw OR HGD:ti,ab,kw OR CIS:ti,ab,kw OR SCC:ti,ab,kw))  AND  ('radiofrequency catheter ablation'/exp) OR (radiofrequency:ti,kw,ab AND (ablation:ti,kw,ab OR energy:ti,kw,ab)) OR RFA:ti,kw,ab OR BARRX:ti,kw,ab |
| **Cochrane clinical trial search strategy** | | |
| Population / Interventions | #1 | (Esophagus OR Esophageal OR Oesophagus OR Oesophageal) AND (Cancer OR Neoplasm OR Malignancy OR Carcinoma OR Dysplasia OR Dysplastic OR Squamous OR LGD OR HGD OR CIS OR SCC) in Title Abstract Keyword  AND  (Radiofrequency AND (Ablation OR Energy)) OR RFA OR BARRX in Title Abstract Keyword |

**Supplementary Table 3. Narration of Enrolled Cohort Trials**

| **First author** | **Year** | **Country (No. of centers)** | **Sample size** | **Study design** | **Inclusion criteria** | **Outcome measures** |
| --- | --- | --- | --- | --- | --- | --- |
| **van Vilsteren FG, et al.^1^** | 2011 | Netherlands (2) | 13 | Prospective case series | Paris classification type 0-IIa to 0-IIc HGIN (12)/ ESCC (1) | - EoT histological CR  - 2-months histological CR  - Acute and late postoperative adverse events |
| **Becker V, et al.^2^** | 2011 | Germany (1) | 6 | Retrospective case series | multifocal ESCC (6) | NA |
| **Haidry RJ, et al.^3^** | 2013 | United Kingdom (8) | 20 | Prospective cohort study | HGIN (12)/ ESCC (8) | - 12-months histological CR  - 3-months histological CR  - Acute and late postoperative adverse events |
| **He S, et al.^4^** | 2015 | China (1) | 96 | Prospective cohort study | 3-12cm MGIN (45)/HGIN (42)/ early flatten ESCC (9) | - 12-months histological CR  - 3-months histological CR  - Acute and late postoperative adverse events |
| **Wang WL, et al.^5^** | 2015 | Taiwan (1) | 65 | Retrospective comparative cohort study | > 50% circumference and > 3cm flatten HGIN (29)/ ESCC (36) | - 12-months histological CR  - 1-months histological CR  - Acute and late postoperative adverse events |
| **Wang WL, et al.^6^** | 2018 | Taiwan (1) | 35 | Retrospective cohort study | > 50% circumference and > 3cm flatten HGIN (25)/ ESCC (10) | - 12-months histological CR  - 1-months histological CR  - Acute and late postoperative adverse events |
| **Yu X, et al.^7^** | 2019 | China (1) | 78 | Retrospective cohort study | 3-12cm MGIN (39)/HGIN (33)/ early flatten ESCC (6) | - 60-months histological CR  - Late postoperative adverse events |
| **Chou YP, et al.^8^** | 2022 | Taiwan (1) | 35 | Retrospective cohort study | HGIN (10)/ ESCC (25) | - 12-months histological CR  - 1-months histological CR  - Acute and late postoperative adverse events |
| **Ding Y, et al.^9^** | 2023 | China (1) | 105 | Retrospective cohort study | > 75% circumference MGIN (21)/HGIN (56)/ESCC (28) | - 12-months histological CR  - 1-months histological CR  - Acute and late postoperative adverse events |

HGIN, high-grade squamous intraepithelial neoplasia; ESCC, esophageal squamous cell carcinoma; EoT, End-of-treatment; CR, complete response; NA, not available; MGIN, moderate-grade squamous intraepithelial neoplasia.

**Supplementary Table 4. Technical Characteristics of Enrolled Cohort Trials**

| **First author** | **RFA protocol** | **Endoscopic sedation** | **Follow-up regimen** | **Postoperative care** |
| --- | --- | --- | --- | --- |
| **van Vilsteren FG, et al.** | HALO360 12 J/cm^2^ - cleaning - 12 J/cm^2^ (10)  HALO90 2 x 15 J/cm^2^ - cleaning - 2 x 15 J/cm^2^ for inner esophageal diameter was < 18 mm, or the unstained area < 2 cm and < 50 % of the esophageal circumference (3) | Conscious sedation with midazolam, fentanyl, pethidine, or monitored anesthesia care with propofol | 6- and EoT follow-up | Esomeprazole (40mg PO BID) and sucralfate PO QID for 14 days with acetaminophen, diclofenac, lidocaine for procedural pain |
| **Becker V, et al.** | HALO360 12 J/cm^2^ - cleaning  HALO90 15 J/cm^2^ - cleaning | Conscious sedation with midazolam in combination with propofol | NA | NA |
| **Haidry RJ, et al.** | HALO360 12 J/cm^2^ for multifocal dysplasia  HALO90 12 J/cm^2^ for unifocal dysplasia | NA | 3-, 6-, 9-, and 12-months follow-up | PPIs PO BID with soluble co-codamol for discomfort post procedure |
| **He S, et al.** | HALO360 1-2 x 10-12 J/cm^2^ +/- cleaning  HALO90 3 x 12 J/cm^2^ | NA | 3-, 6-, 9-, and 12-months follow-up | High-dose PPIs for 1 month |
| **Wang WL, et al.** | HALO360 12 J/cm^2^ - cleaning - 12 J/cm^2^ (18) | Conscious sedation or anesthesia with midazolam, fentanyl, or propofol | 1-, 3-, 6-, and every 6-months follow-up | Esomeprazole (40mg PO QD) and sucralfate suspension PO QID for 1 month with acetaminophen and narcotic PRN |
| **Wang WL, et al.** | HALO360 12 J/cm^2^ - cleaning - 12 J/cm^2^ (35) | NA | 1-, 3-, 6-, and every 6-months follow-up | Esomeprazole (40mg PO QD) and sucralfate suspension PO QID for 1 month |
| **Yu X, et al.** | HALO360 1-2 x 10-12 J/cm^2^ +/- cleaning  HALO90 3 x 12 J/cm^2^ | NA | Annual follow-up | NA |
| **Chou YP, et al.** | HALO360 12 J/cm^2^ - cleaning - 12 J/cm^2^ (4) | NA | 1-, 3-, 6-, 9-, 12-, and every 6-months follow-up | NA |
| **Ding Y, et al.** | HALO360 12 J/cm^2^ - cleaning - 12 J/cm^2^ or 12 J/cm^2^ - cleaning | NA | 1-, 3-, 6-, and every 6-months follow-up | NA |

EoT, End-of-treatment; PO, per os; BID, bis in die; QID, quater in die; NA, not available; PPIs, proton pump inhibitors; PRN, pro re nata.

**Supplementary Table 5. Outcomes Characteristics of Enrolled Cohort Trials**

| **First author** | **Mean age (years)** | **Male (%)** | **Previous ER (%)** | **Mean length of unstained lesions (cm)** | **Outcome measures** | **Intervention group 1** | | |
| --- | --- | --- | --- | --- | --- | --- | --- | --- |
|  |  |  |  |  |  | Event/Total | | ITT (%) |
| **van Vilsteren FG, et al.** | 65 | 61.5 | 69.2 | 5.0 | 2-months complete response | 13/13 | | 100 |
|  |  |  |  |  | EoT complete response | 13/13 | | 100 |
|  |  |  |  |  | Acute adverse events | 4/13 | | 30.8 |
|  |  |  |  |  | Late adverse events | 2/13 | | 15.4 |
|  |  |  |  |  | Bleeding/Laceration/Perforation/  Stricture | 1/2/1/2 | | |
| **Becker V, et al.** | 62 | 100 | 66.7 | NA | NA | NA | | |
| **Haidry RJ, et al.** | 71.6 | 20 | 25 | 6.1 | 3-months complete response | 16/20 | | 80 |
|  |  |  |  |  | 12-months complete response | 10/20 | | 50 |
|  |  |  |  |  | Acute adverse events | 3/20 | | 15 |
|  |  |  |  |  | Late adverse events | 4/20 | | 20 |
|  |  |  |  |  | Bleeding/Laceration/Perforation/  Stricture | 2/1/0/4 | | |
| **He S, et al.** | 59.9 | 54 | 0 | 6.5 | 3-months complete response | 70/96 | | 72.9 |
|  |  |  |  |  | 12-months complete response | 81/96 | | 84.4 |
|  |  |  |  |  | Acute adverse events | 4/96 | | 4.2 |
|  |  |  |  |  | Late adverse events | 20/96 | | 20.8 |
|  |  |  |  |  | Bleeding/Laceration/Perforation/  Stricture | 0/4/0/20 | | |
| **Wang WL, et al.** | 52.8 | 95.4 | 0 | 6.1 | 1-months complete response | RFA | 14/18 | 77.8 |
|  |  |  |  |  |  | ESD | 42/47 | 89.4 |
|  |  |  |  |  | 12-months complete response | RFA | 17/18 | 94.4 |
|  |  |  |  |  |  | ESD | 46/47 | 97.9 |
|  |  |  |  |  | Acute adverse events | RFA | 0/18 | 0 |
|  |  |  |  |  |  | ESD | 4/47 | 8.5 |
|  |  |  |  |  | Late adverse events | RFA | 4/18 | 22.2 |
|  |  |  |  |  |  | ESD | 16/47 | 34 |
|  |  |  |  |  | Bleeding/Laceration/Perforation/  Stricture | RFA | 0/0/0/4 | |
|  |  |  |  |  |  | ESD | 1/1/2/16 | |
| **Wang WL, et al.** | 52.3 | 97 | 0 | 7.2 | 1-months complete response | 25/35 | | 71.4 |
|  |  |  |  |  | 12-months complete response | 32/35 | | 91.4 |
|  |  |  |  |  | Acute adverse events | 3/35 | | 11.4 |
|  |  |  |  |  | Late adverse events | 5/35 | | 14.3 |
|  |  |  |  |  | Bleeding/Laceration/Perforation/  Stricture | 2/1/0/5 | | |
| **Yu X, et al.** | 59.6 | 53 | 0 | 6.1 | 60-months complete response | 67/78 | | 85.9 |
|  |  |  |  |  | Late adverse events | 20/78 | | 25.6 |
|  |  |  |  |  | Stricture | 20 | | |
| **Chou YP, et al.** | 58.3 | 91.4 | 0 | 3.6 | 1-months complete response | RFA | NA |  |
|  |  |  |  |  |  | ESD | 29/31 | 93.5 |
|  |  |  |  |  | 12-months complete response | RFA | 1/4 | 25 |
|  |  |  |  |  |  | ESD | 31/31 | 100 |
|  |  |  |  |  | Acute adverse events | RFA | 0/4 | 0 |
|  |  |  |  |  |  | ESD | 2/31 | 6.5 |
|  |  |  |  |  | Late adverse events | RFA | 1/4 | 25 |
|  |  |  |  |  |  | ESD | 1/31 | 3.2 |
|  |  |  |  |  | Bleeding/Laceration/Perforation/  Stricture | RFA | 0/0/0/1 | |
|  |  |  |  |  |  | ESD | 1/0/1/1 | |
| **Ding Y, et al.** | 67.3 | 75.2 | 0 | 9.4 | 1-months complete response | RFA | 38/45 | 84.4 |
|  |  |  |  |  |  | ESD | 51/60 | 85 |
|  |  |  |  |  | 12-months complete response | RFA | 43/45 | 95.6 |
|  |  |  |  |  |  | ESD | 58/60 | 96.7 |
|  |  |  |  |  | Acute adverse events | RFA | 1/45 | 2.2 |
|  |  |  |  |  |  | ESD | 2/60 | 3.3 |
|  |  |  |  |  | Late adverse events | RFA | 15/45 | 33.3 |
|  |  |  |  |  |  | ESD | 36/60 | 60 |
|  |  |  |  |  | Bleeding/Laceration/Perforation/  Stricture | RFA | 1/0/0/15 | |
|  |  |  |  |  |  | ESD | 1/0/1/36 | |

ER, endoscopic resection; ITT, intention-to-treat; EoT, End-of-treatment; NA, not available; RFA, radiofrequency ablation; ESD, endoscopic submucosal dissection.

**Supplementary Table 6. Risk of Bias for Cross sectional Trials by Newcastle-Ottawa Scale Tool**

| **Author** | **Year** | **Selection** | **Comparability** | **Outcome** | **Score** |
| --- | --- | --- | --- | --- | --- |
| **van Vilsteren FG, et al.** | **2011** | 3 | 0 | 2 | 5 |
| **Haidry RJ, et al.** | **2013** | 3 | 0 | 2 | 5 |
| **He S, et al.** | **2015** | 3 | 0 | 2 | 5 |
| **Wang WL, et al.** | **2015** | 4 | 1 | 2 | 7 |
| **Wang WL, et al.** | **2018** | 3 | 0 | 2 | 5 |
| **Yu X, et al.** | **2019** | 3 | 0 | 2 | 5 |
| **Chou YP, et al.** | **2022** | 4 | 0 | 2 | 6 |
| **Ding Y, et al.** | **2023** | 4 | 0 | 2 | 6 |
|  |  | | | | |

**Supplementary Figure 1A. Pooled Incidence of 12-month Histological Complete Remission after Endoscopic Radiofrequency Ablation (Male percentage > 50%)**

**Supplementary Figure 1B: Pooled Incidence of 3-month Histological Complete Remission after Endoscopic Radiofrequency Ablation (Male percentage > 50%)**

**Supplementary Figure 1C: Pooled Incidence of Acute Postoperative Adverse Events after Endoscopic Radiofrequency Ablation (Male percentage > 50%)**

**Supplementary Figure 1D: Pooled Incidence of Late Postoperative Adverse Events after Endoscopic Radiofrequency Ablation (Male percentage > 50%)**

**
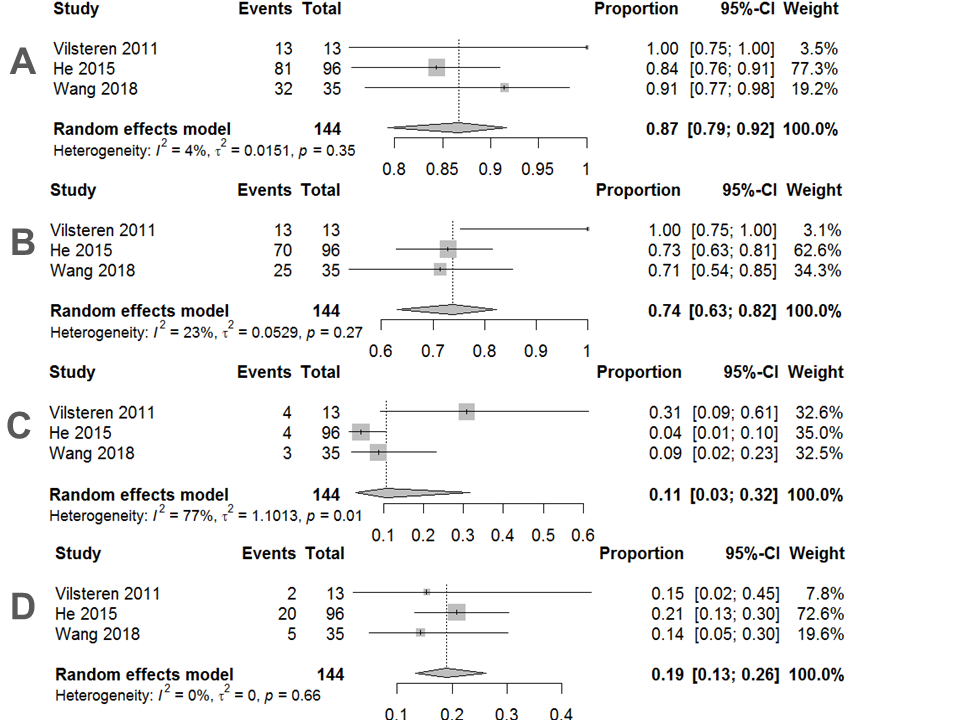
**

**Supplementary References**

1. van Vilsteren FG, Alvarez Herrero L, Pouw RE, ten Kate FJ, Visser M, Seldenrijk CA, van Berge Henegouwen MI, Weusten BL, Bergman JJ. Radiofrequency ablation for the endoscopic eradication of esophageal squamous high grade intraepithelial neoplasia and mucosal squamous cell carcinoma. Endoscopy. 2011; 43: 282-290.
2. Becker V, Bajbouj M, Schmid RM, Meining A. Multimodal endoscopic therapy for multifocal intraepithelial neoplasia and superficial esophageal squamous cell carcinoma - a case series. Endoscopy. 2011; 43: 360-364.
3. Haidry RJ, Butt MA, Dunn J, Banks M, Gupta A, Smart H, Bhandari P, Smith LA, Willert R, Fullarton G, John M, Di Pietro M, Penman I, Novelli M, Lovat LB. Radiofrequency ablation for early oesophageal squamous neoplasia: outcomes form United Kingdom registry. World J Gastroenterol. 2013; 19: 6011-6019.
4. He S, Bergman J, Zhang Y, Weusten B, Xue L, Qin X, Dou L, Liu Y, Fleischer D, Lu N, Dawsey SM, Wang GQ. Endoscopic radiofrequency ablation for early esophageal squamous cell neoplasia: Report of safety and effectiveness from a large prospective trial. Endoscopy. 2015; 47: 398-408.
5. Wang WL, Chang IW, Chen CC, Chang CY, Mo LR, Lin JT, Wang HP, Lee CT. Radiofrequency ablation versus endoscopic submucosal dissection in treating large early esophageal squamous cell neoplasia. Medicine (Baltimore). 2015; 94: e2240.
6. Wang WL, Chang IW, Chen CC, Chang CY, Tseng CH, Tai CM, Lin JT, Wang HP, Lee CT. Lessons from pathological analysis of recurrent early esophageal squamous cell neoplasia after complete endoscopic radiofrequency ablation. Endoscopy. 2018; 50: 743-750.
7. Yu X, van Munster SN, Zhang Y, Xue L, Fleischer DE, Weusten BLAM, Lu N, Dawsey SSM, Bergman JJGHM, Wang G. Durability of radiofrequency ablation for treatment of esophageal squamous cell neoplasia: 5-year follow-up of a treated cohort in China. Gastrointest Endosc. 2019; 89: 736-748.e2.
8. Chou YP, Tai WC, Lu LS, Yao CC, Wu KL, Chuah SK, Lin CY. Endoscopic submucosal dissection and radiofrequency ablation for patients with flat-type esophageal squamous cell neoplasia. Adv Dig Med. 2022; 9: 153-160.
9. Ding Y, Liu Y, Lei S, Zhang W, Qian Q, Zhao Y, Shi R. Comparison between ESD and RFA in patients with total or near-total circumferential early esophageal squamous cell neoplasia. Surg Endosc. 2023 Jun 15.
